# Mutations Causing Mild or No Structural Damage in Interfaces of Multimerization of the Fibrinogen γ-Module More Likely Confer Negative Dominant Behaviors

**DOI:** 10.3390/ijms21239016

**Published:** 2020-11-27

**Authors:** Emanuele Bellacchio

**Affiliations:** Area di Ricerca Genetica e Malattie Rare, Bambino Gesù Children’s Hospital, IRCCS, Piazza Sant’Onofrio 4, 00165 Rome, Italy; Emanuele.Bellacchio@OPBG.net

**Keywords:** protein-protein interactions, fibrinogen storage disease, negative dominant mutation, autosomal recessive mutation, folding free energy change

## Abstract

Different pathogenic variants in the same protein or even within the same domain of a protein may differ in their patterns of disease inheritance, with some of the variants behaving as negative dominant and others as autosomal recessive mutations. Here is presented a structural analysis and comparison of the molecular characteristics of the sites in fibrinogen γ-module, a fibrinogen component critical in multimerization processes, targeted by pathogenic variants (HGMD database) and by variants found in the healthy population (gnomAD database). The main result of this study is the identification of the molecular pathogenic mechanisms defining which pattern of disease inheritance is selected by mutations at the crossroad of autosomal recessive and negative dominant modalities. The observations in this analysis also warn about the possibility that several variants reported in the non-pathogenic gnomAD database might indeed be a hidden source of diseases with autosomal recessive inheritance or requiring a combination with other disease-causing mutations. Disease presentation might remain mostly unrevealed simply because the very low variant frequency rarely results in biallelic pathogenic mutations or the coupling with mutations in other genes contributing to the same disease. The results here presented provide hints for a deeper search of pathogenic mechanisms and modalities of disease inheritance for protein mutants participating in multimerization phenomena.

## 1. Introduction

**Figure 1:**
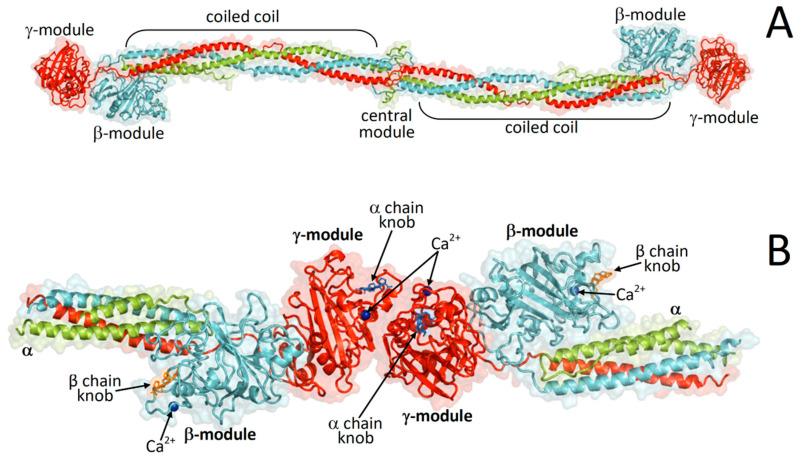
Structures of the hexameric fibrinogen (Aα, Bβ, γ)_2_ and fragment double-D. (**A**) Crystal structure of fibrinogen [1]; (**B**) crystal structure of fragment double-D from human fibrin [2].

Fibrinogen is a secretory glycoprotein complex with a molecular mass of about 340 kDa produced in the liver and resulting from the homodimerization of a heterotrimer composed of the polypeptide chains Aα (*FGA* gene), Bβ (*FGB* gene), and γ (*FGG* gene) with the formal formula (Aα, Bβ, γ)_2_. In each heterotrimer, the Aα, Bβ and γ chains are held together by a triple-helical coiled-coil that links the central nodule, which results from the hexameric assembly of the N-terminal extremities of all six chains to the αC region and the β- and γ-modules. Several inter-chain disulfide bonds stabilize the triple-helical coiled-coil and covalently links the two heterotrimers at the central module. The hexameric fibrinogen assembly has a rod-like shape with each of two extremities (forming the C-terminal portions of the D regions) composed by the β- and γ-module at the C-termini of the respective fibrinogen chains (Figure 1).

Fibrinogen is crucial in coagulation, and in the final phase of clot formation, upon thrombin activation, fibrin monomers form and spontaneously aggregate to form fibrils and subsequently the clot [3]. Genetic changes, mostly point mutations, cause either afibrinogenemia, hypofibrinogenemia, or dysfibrinogenemia. The extent of hypofibrinogenemia is usually related to the heterozygous, homozygous, or compound heterozygous patterns of mutations [4]. Pathological mutations have been observed for each of the three fibrinogen chains. A few mutations in the gamma gene (*FGG*) cause intracellular aggregation and plasma deficiency [5], a condition named hereditary hypofibrinogenemia with hepatic storage (HHHS) [6]. Differently from all the other hypofibrinogenemias, HHHS is not accompanied by overt coagulation problems but always sets the conditions for a progressive liver disease like in the case of α-1-antitrypsin (*AAT*) deficiency [7].

In this work, I made a protein structure analysis to understand the role in disease and the patterns of disease inheritance of known γ-module missense variants. The γ-module is interesting to examine because of its structurally characterized network of interactions; it interacts with the β-module within each Aα-Bβ-γ heterotrimer of the hexameric (Aα, Bβ, γ)_2_ fibrinogen, contains the self-association sites in the γ-chain region of each D domain, which participate in fibrin or fibrinogen D:D assembly and necessary for correct end-to-end alignment of polymerizing fibrinogen or fibrin molecules [8,9], includes the γ-γ-cross-linking sites promoting alignment of cross-linking regions for factor XIII- or FXIIIa-mediated transglutamination [10,11,12], and also provides the hole “a” that spontaneously receives the new N-terminus (Gly-Pro-Arg peptide, named “knob”) exposed by fibrinogen α chain upon proteolysis by thrombin during clotting [13,14].

The stability of a protein structure is determined by the protein folding free energy (∆G), which represents the change of the thermodynamic free energy along the conformational path from the unfolded to the folded state. In particular, the difference in the folding free energy change (∆∆G) between a protein mutant and the corresponding wild type allows to estimate the gain or loss of stability of the local structure upon amino acid mutations, hence allowing to infer whether and to which extent mutations can induce structure alterations. Various computational methods exist to predict the ∆∆Gs associated with protein mutations. In this work, the γ chain crystal structure with the best atomic resolution (representing the isolated γ-module) was analyzed to estimate the amount of structurally damaging and biological implications of known pathogenic missense variants as reported in HGMD [15] and of supposedly non-pathogenic missense variants available in gnomAD [16]. The first aim of the analysis was to identify possible differences in the mutation-induced patterns of structural alterations between these two databases characterized by distinct clinical relevance. Subsequently, various cases of representative variants of HGMD and gnomAD characterized by ∆∆Gs significantly deviating from the average trends in these databases were assessed more explicitly by examining the crystallographic structure of fragment double-D from human fibrin. The analysis of the latter structure allows the gathering of information from the context of functional intermolecular interactions exhibited by the γ-module with other fibrinogen chains. This study provides insights into the molecular implications of γ-module mutations and hints in the identification of mechanisms through which pathogenic mutations can be distinguished between those channelling into autosomal recessive or negative dominant patterns of disease inheritance.

## 2. Results

### 2.1. Distribution of HGMD and GnomAD Missense Variants in the γ-Module

**Figure 2:**
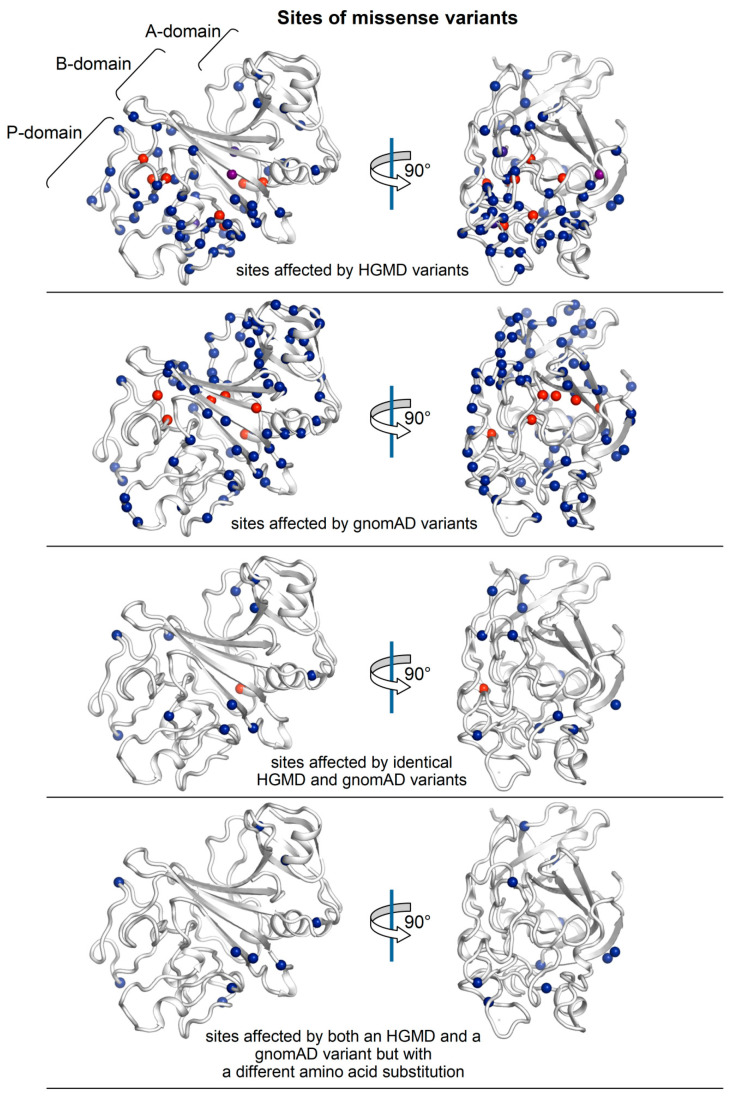
Sites of HGMD and gnomAD missense variants falling in the γ-module of fibrinogen (C*^α^* atoms of affected residues are shown as a sphere in a blue-red gradient according to their distance from the surface of the module: blue, surface-exposed residues; red, residues more in the core of the module).

The HGMD and gnomAD variants falling in the fibrinogen γ-module are distributed almost complementarily (Figure 2). In particular, HGMD variants tend to populate more frequently the C-terminal P-domain (which contains polymerization site), only some fall in the central B-domain and mostly at its external loops with only very few hitting the core of the five-stranded β-sheet. On the other hand, gnomAD variants are more concentrated in the N-terminal A-domain, in particular at its β-sheet and helix, a significant number hits the B domain β-sheet (gnomAD variants hitting this β-sheet also in its core are much more numerous compared to HGMD variants), and relatively few variants fall in the P-domain. Of note, the HGMD and gnomAD databases share a number of identical variants and also residues targeted by mutations but with a different substituting amino acid.

### 2.2. ∆∆G Values of HGMD and GnomAD Missense Variants Localized in the γ-Module

The ∆∆G values of all γ-module missense variants reported in HGMD and gnomAD were calculated using three popular methods based on protein structure analysis, FoldX (v5.0) [17], PoPMuSiC (v3.0) [18], and CUPSAT [19] and shown in Table 1 (HGMD variants) and Table 2 (gnomAD variants). It is worth to notice that the ∆∆Gs of an important number of HGMD variants did not achieve the threshold of significance hence implying that the corresponding amino acid substitutions are predicted not to cause appreciable alterations in the protein structure (Table 1; ∆∆Gs simultaneously predicted by FoldX, PoPMuSiC, and CUPSAT as non-significant for structural changes for the same variant are greyed). On the other hand, several variants in the gnomAD database are predicted with ∆∆Gs suggesting important protein structure alterations (Table 2; the ∆∆Gs simultaneously predicted by FoldX, PoPMuSiC, and CUPSAT as structurally altering for the same gnomAD variant are enclosed in dashed boxes). Both findings are apparently counterintuitive as HGMD, which is supposed to contain pathogenic variants, presents some variants predicted with non-significant protein structure alterations, whereas gnomAD, which excludes variants found in individuals affected by severe pediatric disease and in their first-degree relatives, hence containing only supposedly neutral variants, exhibits several variants predicted to alter the protein structure. Furthermore, and definitely puzzling, as also displayed in Figure 2, the HGMD and gnomAD databases share twelve missense variants (marked with a square in Table 1 and Table 2) and several variants targeting the same residues but with different amino acid substitutions (marked with a triangle in Table 1 and Table 2).

## 3. Discussion

### 3.1. HGMD Variants Predicted to Cause Non-Significant Structural Changes

**Figure 3:**
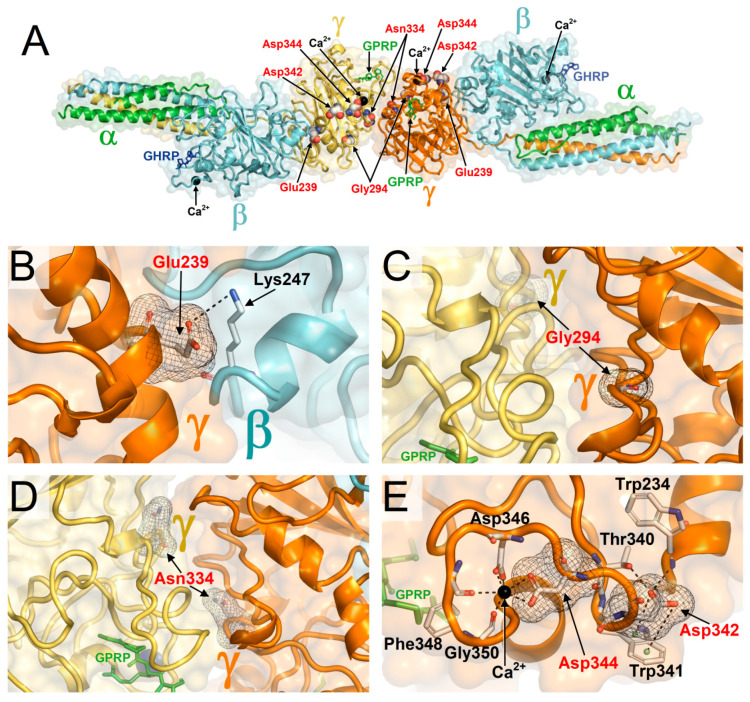
Amino acid residues affected by the missense variants p.Glu239Ala, p.Gly294Glu, p.Asn334Ile, p.Asp342Asn, and p.Asp344Val reported by HGMD and consistently predicted by FoldX, CUPSAT, and PoPMuSiC with ∆∆Gs producing non-significant structural changes in the γ-module. (**A**) The affected sites are shown on the crystal structure of fragment double-D from human fibrin (PDB 1FZC) to highlight their importance in intermolecular interactions. GPRP is an analogue of the α chain knob. GHRP represents the β chain knob. (**B**) Detailed view of the site affected by the p.Glu239Ala variant. The wild type glutamic acid does not contribute significantly to the γ-module fold but is critical for its intermolecular interaction with the β chain via a salt-bridge (dotted lines) with Lys247 in the latter protein. (**C**) Site affected by the p.Gly294Glu variant. The wild-type glycine has no important role in the γ-module fold but is located at the D-D interaction interface which is altered by the non-conservative replacement with glutamic acid. (**D**) Site of the p.Asn334Ile variant. The hydrophilic wild type asparagine is located at the D-D interaction interface; its replacement with the hydrophobic isoleucine alters the properties of the D-D interface. (**E**) p.Asp342Asn and p.Asp344Val variants. Asp342 is engaged in a number of intramolecular interactions (dotted lines), which might be at least partially maintained by the quite conserved replacement with an asparagine. The residue is also close to the Ca^2+^ ion cofactor binding site. Being the affected site at the γ-module surface, it might not cause a severe misfolding. However, this site is not distant from the bound β chain and therefore p.Asp342Asn might affect the interactions with this protein. The p.Asp344Val variant affects an aspartic residue whose carboxylic side chain coordinates the Ca^2+^ ion, and this interaction is lost upon the non-conservative replacement with a valine. In this case, the localization of the change on the surface might not lead to the major unfolding of the γ-module but its intermolecular interactions with other fibrinogen chains can be altered.

The result that HGMD variants on average present ∆∆G values indicating protein structure destabilization satisfy the expectation that pathogenic variants could be more detrimental compared to variants found in the healthy population. However, for several γ-module HGMD variants FoldX, CUPSAT, and PoPMuSiC predict ∆∆G values that do not achieve the threshold of significance (Table 1) hence implying very small or absent structural changes in the protein. In particular, structurally non-significant ∆∆Gs are consistently yielded by the three methods for p.Glu239Ala, p.Gly294Glu, p.Asn334Ile, p.Asp342Asn, and p.Asp344Val (greyed ∆∆Gs in Table 1). This firstly suggests that predicted near-to-zero ∆∆Gs cannot be used as a unique criterion for the identification of neutral mutations since these variants are indeed pathogenic. Then, is also necessary to understand whether the lack of correlation between the pathogenicity of these variants and the predicted minimal/absent structural alterations is due to inaccuracy of the ∆∆G predictions or to other factors that were not considered. Thus, the affected sites were also explicitly examined in the available crystal structures. While ∆∆G calculations were carried out, for increased reliability, on the γ-module crystal structure with best atomic resolution, which was available in the monomeric protein form, the regions targeted by mutations were inspected in the biologically more relevant context of the intermolecular interactions exhibited by the γ-module within the crystal structure of fragment double-D from human fibrin (Figure 3A).

A first observation is that these mutations occur at sites on the γ-module surface. This justifies the predicted near-to-zero ∆∆Gs, as residues exposed on the protein surface usually are less determinant in protein folding compared to residues in the core of the protein. Another observation is that all variants hit or fall in proximity to sites where the γ-module is engaged in intermolecular interactions with other fibrinogen chains. Being the binding regions finely designed for the proper association with the other proteins, their function is highly sensitive to amino acid replacements, even if these produce little or no effects on the fold of the monomeric γ-module. In particular, the residues affected by variants p.Glu239Ala (Figure 3B), p.Gly294Glu (Figure 3C), and p.Asn334Ile (Figure 3D) do not present intramolecular interactions important for the γ-module fold but are located at the D-D dimer interface and therefore are expected to alter homodimerization. The aspartic acid affected by p.Asp342Asn is near the Ca^2+^ cofactor binding site and the aspartic acid hit by p.Asp344Val directly coordinates this Ca^2+^ ion, thus these two mutations have a functional impact on the Ca^2+^ binding region. However, they occur at a peripheral position on the γ-module surface, not in its core (Figure 3D), thus they possibly cause local structural changes but not severe misfolding. Being not distant from the bound β chain (Figure 3A) the interactions with this protein might become defective. Thus, all these HGMD missense variants with consistently predicted near-to-zero ∆∆Gs appear to bear only small or mild and localized γ-module structural alterations, thus not expected to induce full destabilization and loss of function of the γ chain. However, the mutations occur at or near binding regions and likely cause defective assemblies with the other fibrinogen chains. Taken together, these results indicate that the calculations of folding free energy changes on the unbound γ-module plausibly predicted that these group of HGMD variants can be somehow tolerated but this information alone is not sufficient to exclude other pathogenic mechanisms, specifically defects in protein-protein interactions (Table 1 shows the binding status for all γ-module residues affected by HGMD missense mutations as determined in fragment double-D). A corollary is that the tolerability of the local protein structure to amino acid changes does not necessarily imply the absence of pathogenic effects. Indeed, the cases examined above suggest that the capability of a protein to structurally and functionally tolerate amino acid replacements can paradoxically become itself a subtle cause of pathogenicity. Specifically, this can happen when a) the protein variant does not unfold sufficiently to be recognized as defective and driven to degradation by cellular mechanisms of protein quality check, and b) the variant also retains the capability of its designed functional interactions with other protein chains flawing the arrangement and functions of quaternary structures and/or triggering anomalous protein aggregations. In such instances, small structural defects can propagate and amplify dramatically if incorporated in the higher structural organization levels and functions of protein multimers. Imperfect or undue recruitments of other proteins and the formation of corrupted or de novo protein assemblies underlie negative dominant mechanisms. A protein such as γ fibrinogen that undergoes a complex, finely regulated, and irreversible polymerization can be particularly exposed to such risk. Indeed, in previous works, we proposed mechanisms explaining how mutations affecting the interface of homodimerization of fibrinogen γ might lead to HHHS [20]. We also showed how fibrinogen defects can trigger storage disease and plasma deficiency not only of fibrinogen but also of non-fibrinogen proteins such as apolipoprotein B [21]. We in fact presented a case of a child with hypofibrinogenemia due to the Aguadilla mutation and severe hypobetalipoproteinaemia demonstrating that both fibrinogen and apolipoprotein B accumulated in the same endoplasmic reticulum inclusions despite the latter protein was not mutated.

### 3.2. GnomAD Variants Predicted to Be Structurally Damaging

**Figure 4:**
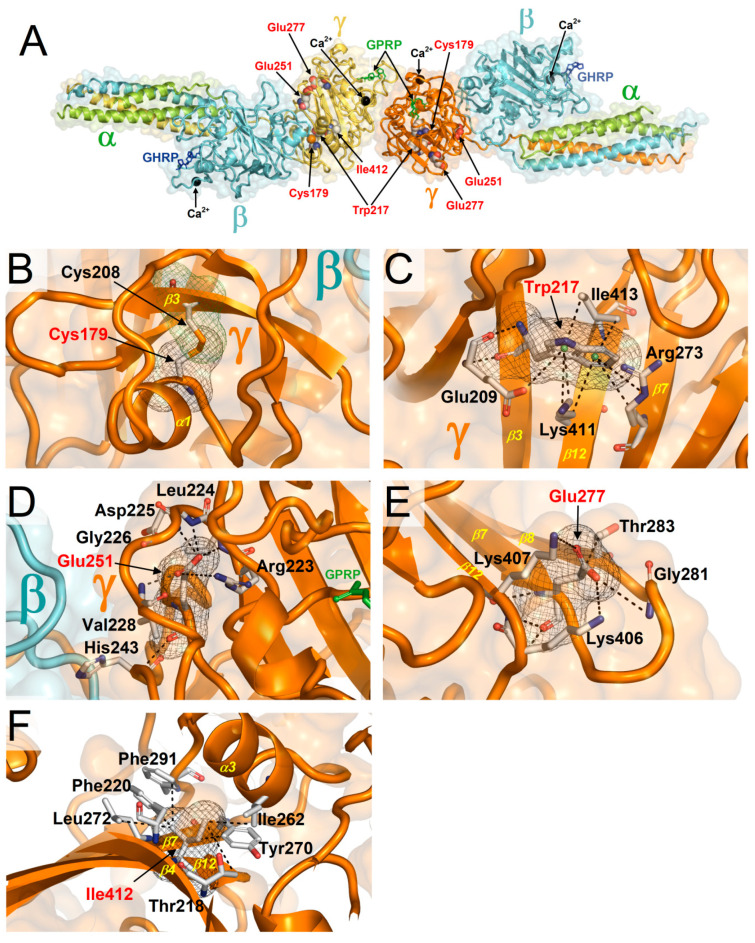
Amino acids affected by the missense variants p.Cys179Phe, p.Trp217Gly, Glu251Gly, p.Glu277Gly, and p.Ile412Thr selected among those reported by gnomAD and consistently predicted by FoldX, CUPSAT, and PoPMuSiC to destabilize significantly the γ-module structure. (**A**) The wild type residues are shown on the crystal structure of fragment double-D from human fibrin (PDB 1FZC). GPRP is an analog of the α chain knob. GHRP represents the β chain knob. (**B**) Detailed view of the site of the p.Cys179Phe variant. The wild type cysteine 179 forms a disulfide bond with cysteine 208 in the core of the γ-module. The non-conserved replacement by phenylalanine is expected to cause misfolding. (**C**) The p.Trp217Gly replacement is also non-conserved, it causes the loss of several intramolecular interactions holding together various β strands in the γ-module core and is expected to cause misfolding. (**D**) The non-conserved p.Glu251Gly substitution causes the loss of multiple intramolecular interactions and is expected to cause severe structural changes in the γ-module. (**E**) The non-conserved p.Glu277Gly replacement disrupts multiple intramolecular interactions necessary to tighten various β strands and is predicted to cause important structural alteration. (**F**) The p.Ile412Thr variant affects isoleucine engaged in several hydrophobic interactions contributing to the γ-module fold. The non-conserved replacement with a threonine is expected to misfold the module. In the detailed views, the interactions between the affected wild type residues and surrounding residues contributing to the fold of the γ-module, are indicated by dotted lines.

FoldX, CUPSAT, and PoPMuSiC consistently compute that 25 out of 89 (28%) gnomAD missense variants hitting the γ-module are associated with ∆∆Gs significant for structural alterations (Table 2, ∆∆Gs enclosed in dashed boxes). Although not always true, important defects in protein structures can be the underlying cause of diseases. Thus, it was intriguing to see that so many variants in the gnomAD database, which is supposed to represent the healthy population, were predicted as strongly destabilizing the γ-module structure. Then, it was interesting to understand why mutations predicted to significantly alter the protein structure can be so divergent in their outcomes on health, as in some cases they behave as neutral mutations (gnomAD variants with high ∆∆Gs) or as pathogenic mutations (HGMD variants). In the case of the apparently anomalous gnomAD variants, an explicit analysis of the atomic configurations around the affected sites was made. Given the high number of the gnomAD variants characterized by remarkably high ∆∆Gs, the analysis was limited to few but representative cases: p.Cys179Phe, p.Trp217Gly, Glu251Gly, p.Glu277Gly, and p.Ile412Thr. The affected wild-type residues are illustrated in the crystal structure of the D-D dimer (Figure 4A), and the roles and expected effects of the amino acid replacements will now be commented on more in detail.

The p.Cys179Phe substitution disrupts the disulfide bond between Cys179 and Cys208, which in the wild type protein tighten α-helix 1 to β-strand 3 and contribute to the fold of the γ-module core (Figure 4B). This substitution should cause γ-module misfolding and loss of functions. Indeed, indications that Cys179 is critical for γ-module function can be inferred from another variant affecting the same residue, p.Cys179Arg, already known as pathogenic (Table 1). The p.Trp217Gly variant affects a tryptophan in the core of the γ-module that is engaged in multiple interactions holding together β-strand 3, β-strand 7, and β-strand 12 (Figure 4C). The replacing glycine, which lacks the side chain, cannot provide the same intramolecular stabilization as the wild type tryptophan residue and cannot preserve the γ-module fold and functions. The p.Glu251Gly variant is another case were the multiple interactions sustained by the wild type residue, a glutamic acid, cannot be replaced by glycine (Figure 4D). This non-conservative change is expected to cause major structural alteration at the γ-module region of interaction with the β-module. The p.Glu277Gly affects a glutamic acid that provides several intramolecular interactions shaping the loop between β-strand 7 and β-strand 8 and also tightening these two strands to β-strand 12 (Figure 4E). In this case, these interactions are also lost upon the replacement with a glycine, which allows one to foresee important unfolding. Finally, the p.Ile412Thr variant hits isoleucine characterized by several hydrophobic interactions that hold together β-strand 4, β-strand 7, β-strand 12, and α-helix 3 thus contributing critically to the fold of the γ-module core. The non-conservative replacement of this isoleucine with a threonine is also expected to cause an important structural alteration in the γ-module. Thus, the visual inspection on experimental γ-module structures supports the destabilizing ∆∆Gs of these gnomAD variants as consistently predicted by FoldX, CUPSAT, and PoPMuSiC. In all the gnomAD cases here examined, a severe misfolding of the γ-module and complete loss of its functions is the likely outcome. Structural alterations only of the moderate entity and confined near the affected sites so that they would warrant a somehow normal γ-module functioning are not plausible. In fact, the non-conserved amino acid substitutions in these particular locations with critical structural importance cannot occur without important conformational changes, which, in such a small-sized globular structure, are easily relayed and extended to other γ-module regions severely impairing its functions. Thus, these gnomAD variants are candidates as pathogenic variants. Below I explain why variants strongly altering the protein structure do not always end up in noticeable diseases.

### 3.3. Rationalization of the Missense Variants Shared by the HGMD and GnomAD Databases

**Figure 5:**
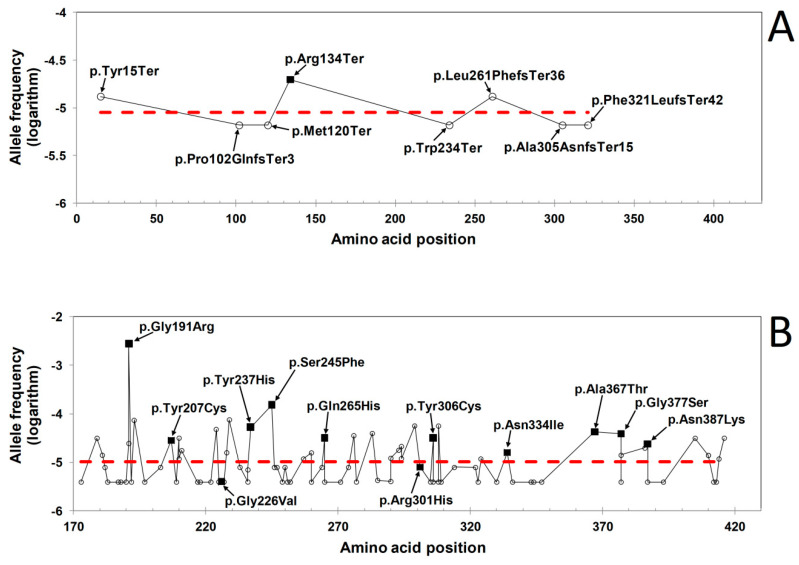
Allele frequency (in base-10 logarithm scale) of gnomAD variants. (**A**) Truncating and frameshift variants. (**B**) Missense variants. The variants simultaneously reported by gnomAD and HGMD are indicated by closed squares, variants reported only by gnomAD are indicated with open circles. The horizontal dashed red line represents the averaged logarithms of allele frequencies of all variants displayed in the individual graphs. As a trend, it can be noticed that in both graphs, variants simultaneously reported by gnomAD and HGMD tend to be more frequent than variants recorded exclusively in gnomAD.

GnomAD includes many missense variants in the γ-module expected to severely impair its structure and function. To dissipate doubts that a significant number of them can be pathogenic suffices to notice that gnomAD shares twelve missense variants with the pathogenic HGMD database: p.Gly191Arg, p.Tyr207Cys, p.Gly226Val, p.Tyr237His, p.Ser245Phe, p.Gln265His, p.Arg301His, p.Tyr306Cys, p.Asn334Ile, p.Ala367Thr, p.Gly377Ser, and p.Asn387Lys. Most of these variants imply non-conservative amino acid changes and critically affect the protein structure/function. The case of p.Asn334Ile is illustrated in Figure 3D, but some of these variants have been functionally characterized and described in the literature. gnomAD and HGMD also contain additional variants affecting the same amino acid residues but with different amino acid replacements (gnomAD and HGMD variants that are identical or that affect the same residue but with different substituting residues are marked in Table 1 and Table 2). The fibrinogen variants simultaneously included in both HGMD and gnomAD can be rationalized assuming that their impact on the protein structure is sufficient to induce its degradation or to reduce, at least partially, its ability to bind other proteins thus limiting negative dominant effects such as defective polymerization or aspecific aggregations. This also requires that haploinsufficiency is absent or only modest. This last point can be confirmed by the truncating or frameshift mutations in the fibrinogen γ chain currently available in gnomAD and all reported exclusively in heterozygosity: p.Tyr15Ter, p.Pro102GlnfsTer3, p.Met120Ter, p.Arg134Ter, p.Trp234Ter, p.Leu261PhefsTer36, p.Ala305AsnfsTer15, p.Phe321LeufsTer42. These mutations are clearly problematic for the γ chain functions as they range from null mutations (the most N-terminal variants) to mutations that delete totally or to a great extent at least the γ-module sequence, which spans residues 174–415 ca. (numbering from the initiator methionine of fibrinogen γ chain: UniProt code P02679). Among the above mutations, those that abrogate the functions of fibrinogen are candidates as causative a disease such as afibrinogenemia, which requires homozygosity or compound heterozygosity of null mutations. It is worth to mention that p.Arg134Ter, which is unique among the above truncating and frameshift gnomAD variants also reported in HGMD, was found in homozygosity in ten afibrinogenemic patients born after consanguineous marriages [22]. Interestingly enough, p.Arg134Ter is the variant with the highest allele frequency within the truncating and frameshift gnomAD variants (Figure 5A). Another interesting observation can be made examining the allele frequencies of the missense variants in gnomAD. In this case, it also happens that the most frequent gnomAD missense variant, p.Gly191Arg (allele frequency 2.77E-03), is also reported in HGMD. The second most frequent gnomAD missense variant, p.Ser245Phe (allele frequency of 1.52E-04), is also present in HGMD. Furthermore, most of the other gnomAD missense variants that happened to be simultaneously reported by HGMD are also distributed on the higher frequency side compared to the missense variants represented exclusively in the gnomAD database (Figure 5B). It, therefore, appears that some of the gnomAD variants were eventually characterized as pathogenic simply because they occur less rarely than most of the gnomAD variants. This is why potentially pathogenic variants can be found in gnomAD, or, more correctly, their pathogenic nature waits to be discovered.

This study allows one to propose that the gnomAD database contains several potentially pathogenic variants that obey a recessive modality of disease transmission and/or exhibit negative dominant effects in a non-quantitative fashion, or at least not causing overt diseases so that they require additional pathogenic mutations in other fibrinogen chains or other genes and/or particular environmental factors to result in evident or more severe illness. Carriers of such variants with only one defective allele can present physical conditions ranging from normal health to mild and/or difficult to identify disorders, or diseases with late-onset or triggered by environmental factors or trauma, etc. Fortunately, most of the γ-module variants catalogued in gnomAD present very low allele frequency (Table 2) and those that could indeed be pathogenic rarely combine with the same or other pathogenic mutations. However, there are exceptions like p.Arg301His, which is reported in both HGMD and gnomAD databases (in the latter with allele frequency 8.00E–06) and considered a hotspot mutation for dysfibrinogenemia. One of the disorders associated with this variant is thromboembolism whose genetic risk is increased also by p.Gln534Arg in coagulation factor V (factor V Leiden variant), which is also reported in gnomAD and quite common (allele frequency of 9.81E−01). While heterozygous carriers of either one or the other variant are mostly asymptomatic, patients with both heterozygosities manifest thromboembolism [23,24]. Despite fibrinogen γ chain p.Arg301His is a rare pathogenic mutation (its base-10 logarithm frequency is close to the average for the gnomAD fibrinogen γ chain missense variants, Figure 5), this variant can easily combine with the much more common factor V Leiden variant and lead quantitatively to thromboembolism.

Care must be exercised with variants found in the healthy population and believed to represent neutral mutations as a yet unknown fraction of them can be disease-causing mutations.

## 4. Materials and Methods

### Protein Structural Analysis

The structural analysis to determine the regions of binding of the γ-module was performed on the crystal structure of fragment double-D from human fibrin (PDB entry 1FZC). ∆∆G calculations were made with FoldX (v5.0), PoPMuSiC (v3.0), and CUPSAT for one amino acid replacement at a time on the crystal structure of the C-terminal fragment of the fibrinogen gamma chain representing the unbound γ-module with best atomic resolution reported to date (PDB entry 3FIB). Prior to executing FoldX, the PDB repair utility was applied to the crystal structure coordinate file, and calculations were then carried out averaging the ∆∆G results of FoldX over 5 runs for each mutation. ∆∆G calculations with PoPMuSiC and CUPSAT were performed directly on the original PDB structure 3FIB. The stability change thresholds here employed were those tested for the highest accuracy of predictions of the individual FoldX, PoPMuSiC, and CUPSAT methods on sets of experimentally characterized mutations: FoldX, |∆∆G| > 1.0 Kcal/mol [25]; PoPMuSiC, |∆∆G| > 0.5 Kcal/mol [26], and CUPSAT, |∆∆G| > 1.0 Kcal/mol [19]. Protein secondary structure elements were determined with STRIDE [27]. Molecular graphics were made with PyMOL (www.pymol.org). The missense variants of gnomAD were retrieved from the database v2.1.1, the truncating and frameshift variants of gnomAD were collected from the database v3.1. HGMD variants were downloaded from the public version of the database in the first half of May 2020.

## 5. Conclusions

Four main findings emerge from this work regarding useful criteria to discriminate recessive from negative dominant variants in the fibrinogen γ chain.

(a) A number of variants in the healthy population might indeed be pathogenic with an autosomal recessive modality of disease transmission. Variants with such characteristics can especially be those inducing severe protein misfolding leading to the degradation of the protein or at least its inability to recruit the designed interacting fibrinogen protein partners or to undergo undue aggregations, i.e., variants causing full loss of function, or not insinuating into negative domain effects.
(b) Other potentially disease-causing variants found in the healthy population are those with negative dominant effects producing mild and/or difficult to identify disorders and/or late-onset diseases, or disorders caused by specific environmental factors, trauma, etc.
(c) A recurrent observation in this and previous studies is that a number of pathogenic variants hit the protein structure only “softly” but in spots important for protein-protein interactions. Proteins with defects in these critical regions, if not efficiently neutralized by the cellular mechanism of protein degradation or at least by a loss of the protein capacity to bind and recruit other proteins render these variants candidates to be checked as negative dominant mutations. This can be particularly true for proteins engaging in processes of homo- and homo-hetero multimerization.
(d) This study highlights that a significant fraction of gnomAD variants are not neutral (for their co-presence in the HGMD database), and this might be only the tip of the iceberg (as suggested by ∆∆G calculations and protein structural analysis). Care must be exercised for all variants found in the general population and believed as neutral mutations.

Finally, the considerations here presented might help to address the pathogenic mechanisms of many other proteins that, likewise the fibrinogen γ chain, are involved in homo- and hetero-multimerization processes.

## Figures and Tables

**Table 1:** ∆∆G values of γ-module HGMD missense variants.

Variant	∆∆G (Kcal/mol)			Interactions ^a^	Secondary Structure ^b^	%SAS ^c^
	FoldX ^d^	CUPSAT ^d^	PoPMuSiC ^d^			
	3.9 ± 5.4 ^e^	−1.44 ± 3.06 ^e^	1.16 ± 0.80 ^e^			
p.Cys179Arg^▲^	**21.8**	**−9.16**	**2.90**		α-helix	0.0
p.Gly191Arg^■▲^	**4.9**	**−2.77**	**2.63**		β-strand	2.1
p.Phe204Leu	**2.9**	−0.03	**1.25**	Bβ	β-strand	12.2
p.Tyr207Cys^■^	**1.1**	**−4.23**	**2.24**		β-strand	19.8
p.Gly226Val^■^	**6.2**	**−3.98**	**2.35**		coil	14.7
p.Trp234Leu	**2.9**	**−2.80**	**1.95**		α-helix	10.7
p.Tyr237His^■^	**3.1**	**−1.81**	**1.65**		α-helix	0.0
p.Glu239Ala	0.6	−0.28	−0.20	Bβ	α-helix	86.4
p.Ser245Phe^■^	0.5	**−3.67**	**0.78**	Bβ	turn	15.8
p.Trp253Cys	**4.6**	**3.21**	**2.50**	Bβ	β-strand	0.0
p.Asn256Lys	**3.5**	**−5.30**	**0.83**		α-helix	1.0
p.Asn256His	**6.6**	**−1.75**	−0.07		α-helix	1.0
p.Asn256Asp	**1.1**	**−3.27**	**0.76**		α-helix	1.0
p.Lys258Thr	**2.0**	0.20	**0.50**	Bβ	α-helix	39.1
p.Gln265His^■▲^	**8.1**	**−1.17**	0.44		310 helix	3.0
p.Trp279Gly	**2.6**	**−1.64**	**2.80**		turn	19.5
p.Trp279Cys	**2.3**	**−1.11**	**2.15**		turn	19.5
p.Tyr288Cys	**5.8**	**5.07**	**2.90**		β-strand	0.0
p.Gly294Glu^▲^	0.1	0.40	0.20	****γ****	coil	54.5
p.Arg301Ser^▲^	0.8	0.81	**1.34**	**γ**	turn	35.0
p.Arg301His^■^^▲^	**1.7**	−0.79	**0.84**	**γ**, knob	turn	35.0
p.Arg301Cys^▲^	**1.3**	−0.81	**1.26**	**γ**, knob	turn	35.0
p.Thr303Arg	−0.5	−0.74	**0.96**	**γ**	β-strand	43.9
p.Thr303Pro	**2.4**	−0.34	**2.24**	**γ**	β-strand	43.9
p.Ala305Asp^▲^	0.1	**−1.33**	0.32	**γ**	β-strand	41.5
p.Tyr306Cys^■▲^	**1.2**	**−6.61**	**0.81**	**γ**	β-strand	61.0
p.Gly310Arg	**13.5**	**−2.56**	**1.90**		turn	37.7
p.Ala315Val	**4.5**	**1.89**	**0.65**		310 helix	0.0
p.Gly318Val	**15.4**	**−12.81**	**2.48**		coil	0.0
p.Thr331Ala	**1.5**	−0.39	**1.28**	knob	α-helix	7.6
p.His333Tyr	**7.6**	**−5.85**	0.46		turn	0.8
p.Asn334Lys	0.3	**−1.75**	0.69	**γ**	turn	46.6
p.Asn334Thr	**1.5**	**−1.16**	−0.17	**γ**	turn	46.6
p.Asn334Ile^■^	0.5	−0.18	−0.04	**γ**	turn	46.6
p.Gly335Asp	**2.9**	**−4.01**	**1.42**	**γ**	turn	60.7
p.Gly335Cys	**3.1**	−0.13	**1.42**	**γ**	turn	60.7
p.Met336Thr^▲^	**2.3**	0.19	**1.69**	**γ**	bridge	13.0
p.Ser339Asn	**6.0**	**−4.03**	**1.19**		bridge	0.0
p.Ser339Arg	**16.1**	**−1.66**	**0.81**		bridge	0.0
p.Ser339Gly	**1.1**	**−3.53**	**1.32**		bridge	0.0
p.Thr340Ile	**1.8**	−0.83	**0.62**		bridge	0.0
p.Thr340Pro	**2.9**	**−12.46**	**1.79**		bridge	0.0
p.Asp342Asn	0.1	0.32	0.01	proximal to Ca^2+^	turn	40.1
p.Asp342His	**1.1**	−0.77	0.32	proximal to Ca^2+^	turn	40.1
p.Asp342Gly	0.9	0.11	**0.92**	proximal to Ca^2+^	turn	40.1
p.Asp344Gly^▲^	−0.5	−0.45	**0.84**	Ca^2+^	turn	37.3
p.Asp344Val^▲^	0.9	0.00	0.34	Ca^2+^	turn	37.3
p.Asp344Tyr^▲^	0.6	0.30	**0.76**	Ca^2+^	turn	37.3
p.Asn345Lys	**1.6**	**−2.21**	**0.81**	proximal to Ca^2+^	coil	17.1
p.Asn345Asp	**3.8**	−0.74	**0.74**	proximal to Ca^2+^	coil	17.1
p.Asp346Glu	**5.9**	0.07	**1.16**	Ca^2+^	coil	0.0
p.Asp346Gly	**1.6**	**−2.66**	**1.58**	Ca^2+^	coil	0.0
p.Phe348Ile	**5.4**	**−1.51**	**1.23**	knob, Ca^2+^	turn	32.8
p.Phe348Cys	**3.0**	0.26	**1.70**	knob, Ca^2+^	turn	32.8
p.Asn351Ile	**1.6**	0.05	**1.37**	proximal to Ca^2+^	turn	21.0
p.Cys352Ser	**2.9**	**−2.86**	**1.99**	proximal to Ca^2+^	α-helix	0.0
p.Cys352Tyr	**32.3**	**−2.43**	0.81	proximal to Ca^2+^	α-helix	0.0
p.Cys352Phe	**23.1**	**−1.55**	**1.19**	proximal to Ca^2+^	α-helix	0.0
p.Ala353Thr	**4.8**	**−1.04**	**1.09**	proximal to Ca^2+^	α-helix	0.0
p.Gln355Arg	0.5	**1.43**	**0.51**	knob	α-helix	32.1
p.Asp356Val	**−1.8**	**−2.62**	**0.57**	knob	α-helix	0.0
p.Asp356Tyr	**0.5**	**−1.45**	−0.02	knob	α-helix	0.0
p.Ser358Cys	**1.8**	**−7.02**	−0.34		coil	0.8
p.Trp361Arg	**6.4**	**9.09**	**2.81**		coil	0.8
p.Met362Ile	**2.9**	**−6.89**	**1.13**		turn	0.0
p.Asn363Lys	**1.4**	0.32	**1.52**		turn	6.9
p.Ala367Thr^■^	**3.0**	0.39	**1.25**		turn	0.0
p.Ala367Asp^▲^	**6.4**	−0.25	**2.14**		turn	0.0
p.Ala367Val^▲^	**2.2**	0.77	0.43		turn	0.0
p.Asn371Ser	**3.1**	**−1.72**	**1.41**		turn	0.0
p.Asn371Asp	**1.5**	−0.15	**1.59**		turn	0.0
p.Gly372Val	**18.2**	**−3.53**	0.37		turn	4.2
p.Tyr374Cys	**3.1**	−0.33	**1.73**		coil	12.4
p.Gly377Ser^■▲^	**3.1**	−0.55	**1.49**		turn	92.1
p.Tyr380Cys	**4.6**	−0.61	**2.48**		coil	1.2
p.Ala383Thr	0.2	**1.43**	−0.16	proximal to knob	turn	100
p.Ser384Cys	−0.1	**−2.52**	0.12		turn	32.3
p.Asn387Lys^■▲^	−0.3	**2.27**	**0.59**	proximal to knob	turn	83.8
p.Tyr389Asn	**1.5**	**−2.78**	**1.33**	knob	coil	40.1
p.Asp390His	**1.8**	**−1.96**	**0.65**	knob	coil	19.9
p.Asp390Val	**3.1**	**−2.02**	0.37	knob	coil	19.9
p.Asn391Lys	−0.7	**3.20**	**1.34**	proximal to knob	coil	16.1
p.Gly392Ser	**4.6**	**−4.01**	**0.81**		coil	0.0
p.Trp395Leu	**3.1**	0.55	**1.75**		β-strand	0.0
p.Thr397Ile	**1.4**	**−1.35**	**0.58**		turn	21.6
p.Arg401Gly	**2.3**	**−1.00**	**2.19**	knob	turn	28.4
p.Arg401Trp	**2.9**	**3.21**	0.42	knob	turn	28.4
p.Ser404Pro	**6.5**	**−1.34**	**2.20**		coil	0.0
p.Lys406Asn	**1.9**	−0.39	**0.98**		coil	40.3

HGMD amino acid variants and their ∆∆Gs (calculated with FoldX, CUPSAT, and PoPMuSiC on the crystal structure of the C-terminal fragment of the fibrinogen gamma chain monomer, PDB 3FIB), molecular interactions, protein secondary structure, and the solvent-accessible surface of the residues affected by mutations. ^a^ Intermolecular interactions of the wild type residues with functional ligands (Bβ, γ, knob, and Ca^2+^ ion, determined in the crystal structure of fragment double-D from human fibrin, PDB 1FZC). ^b^ Protein secondary structure of the wild type residues (determined on PDB 3FIB). ^c^ Percentage of the solvent-accessible surface of side chains of the wild type residues (determined on PDB 3FIB). ^d^ Structurally significant ∆∆Gs are in bold. ∆∆Gs of given variants are greyed if they do not achieve structural significance in all three methods (FoldX, CUPSAT, and PoPMuSiC). For both FoldX and CUPSAT, mutations with destabilizing and stabilizing effects on protein structure are respectively associated with ∆∆G > 0 and ∆∆G < 0, while for PoPMuSiC is the inverse, i.e., ∆∆G > 0 for stabilizing mutations and ∆∆G < 0 for destabilizing ones. The ∆∆G thresholds above which mutations are assumed to produce significant structure alteration are as follows: for both FoldX and CUPSAT, |∆∆G| > 1.0 Kcal/mol, while for PoPMuSiC, |∆∆G| > 0.5 Kcal/mol (see Materials and Methods for references on these thresholds and the predictive accuracies of the individual methods on experimentally determined mutations). ^e^ Mean and standard deviation of all ∆∆Gs calculated by the individual methods. ^■^ Same variant is also reported in the non-pathogenic gnomAD database. ^▲^ A variant hitting the same residue but with different substituting amino acids is reported in gnomAD.

**Table 2:** ∆∆G values of γ-module gnomAD missense variants.

Variant	∆∆G (Kcal/mol)			Interactions ^a^	Secondary Structure ^b^	%SAS ^c^	Allele Frequency
	FoldX ^d^	CUPSAT ^d^	PoPMuSiC ^d^				
	1.8 ± 2.8 ^e^	−1.45 ± 2.88 ^e^	0.95 ± 0.86 ^e^				
p.Asp173Gly	0.3	**−1.87**	0.44		β-strand	100.0	3.98E−06
p.Cys179Phe^▲^	**21.1**	**−1.07**	**1.00**		α-helix	0.0	3.18E−05
p.Asp181Asn	0.4	−0.91	**0.61**		α-helix	41.8	1.42E−05
p.Ile182Val	0.7	**−2.46**	**1.40**		α-helix	0.0	7.96E−06
p.Ala183Val	2.3	−0.08	**0.58**		α-helix	1.1	3.98E−06
p.Ala187Thr	0.9	**−1.31**	**0.88**		coil	2.2	3.98E−06
p.Lys188Arg	−0.7	**−1.41**	0.04		coil	87.9	3.98E−06
p.Ser190Ile	**2.0**	**−0.74**	0.24		coil	41.2	3.98E−06
p.Gly191Glu^▲^	**5.6**	**−11.52**	**3.17**		β-strand	2.1	2.48E−05
p.Gly191Arg^■^	**5.2**	**−2.77**	**2.63**		β-strand	2.1	2.77E−03
p.Leu192Ile	**2.0**	**1.25**	**0.75**	Bβ	β-strand	10.1	3.98E−06
p.Tyr193His	**3.6**	**−5.10**	**2.69**		β-strand	2.6	7.43E−05
p.Pro197Thr	**4.2**	**−1.91**	**1.49**		turn	0.0	3.98E−06
p.Gln203Lys	0.1	0.59	0.11	Bβ	turn	61.9	7.96E−06
p.Tyr207Cys^■^	**1.0**	**−4.23**	**2.24**		β-strand	19.8	2.83E−05
p.Glu209Lys	−0.7	**−1.11**	**0.86**		β-strand	41.4	3.98E−06
p.Ile210Met	**1.1**	**1.37**	**1.31**		β-strand	0.9	3.19E−05
p.Ile210Ser	**3.7**	0.12	**2.99**		β-strand	0.9	1.19E−05
p.Asp211Asn	**1.2**	−0.54	0.43		turn	50.6	1.77E−05
p.Trp217Gly	**4.9**	**−5.89**	**4.45**		β-strand	7.2	3.98E−06
p.Thr218Ile	−0.8	−0.08	0.00		β-strand	2.8	3.98E−06
p.Lys222Glu	**1.9**	**−1.94**	**0.75**		β-strand	32.2	3.98E−06
p.Leu224Arg	**1.0**	−0.90	**0.66**		coil	20.3	4.83E−05
p.Asp225Tyr	0.6	**−2.57**	**0.50**	Bβ	coil	55.0	4.02E−06
p.Gly226Val^■^	**6.1**	**−3.98**	**2.35**		coil	14.7	4.01E−06
p.Ser227Asn	−0.1	**−1.93**	0.22	Bβ	coil	53.1	4.01E−06
p.Val228Ala	**1.7**	**−3.48**	**2.11**		coil	10.9	1.60E−05
p.Asp229Asn	**1.2**	**−1.07**	0.16		coil	92.6	7.61E−05
p.Asn233Thr	**3.5**	**−1.40**	0.42	Bβ	coil	46.9	7.99E−06
p.Gln236His	0.7	0.10	**0.59**	Bβ	α-helix	54.9	3.99E−06
p.Gln236Arg	0.0	**0.82**	0.31	Bβ	α-helix	54.9	7.10E−06
p.Tyr237His^■^	**3.1**	**−1.81**	**1.65**		α-helix	0.0	5.32E−05
p.Ser245Phe^■^	0.2	**−3.67**	**0.78**	Bβ	turn	15.8	1.52E−04
p.Pro246Arg	**1.8**	−0.26	**0.93**		turn	56.9	7.97E−06
p.Thr247Ala	−0.5	**2.35**	0.44		turn	83.0	7.97E−06
p.Thr249Ala	−0.6	0.54	**0.69**	Bβ	coil	76.8	3.98E−06
p.Thr250Ile	0.4	**−1.59**	−0.04	Bβ	coil	36.7	7.97E−06
p.Glu251Gly	**2.7**	**−3.34**	**1.88**		coil	0.0	3.98E−06
p.Phe252Leu	**2.5**	**−3.86**	**1.60**	Bβ	β-strand	7.8	3.98E−06
p.Glu257Ala	0.8	−0.46	**1.08**		α-helix	50.4	1.19E−05
p.His260Arg	**1.0**	−0.48	**0.52**		α-helix	23.4	1.59E−05
p.His260Asn	0.9	−0.15	**1.24**		α-helix	23.4	3.98E−06
p.Thr264Pro	**5.7**	**−5.49**	**1.66**		α-helix	36.4	7.97E−06
p.Gln265His^■^	**8.8**	**−1.17**	0.44		310 helix	3.0	3.19E−05
p.Gln265Glu^▲^	**3.9**	**−1.05**	**1.00**		310 helix	3.0	3.98E−06
p.Ala271Ser	**1.8**	**−4.60**	**0.63**		β-strand	2.2	3.98E−06
p.Val274Met	0.0	**−8.19**	**1.78**		β-strand	0.0	7.97E−06
p.Leu276Met	0.9	**−9.83**	**1.45**		β-strand	0.5	3.59E−05
p.Glu277Gly	**1.4**	**−2.27**	**1.51**		β-strand	34.1	3.99E−06
p.Thr283Ile	−0.4	**−4.75**	0.27		β-strand	60.8	3.99E−05
p.Thr285Ala	0.4	**−1.32**	**1.40**		β-strand	39.5	4.32E−06
p.Met290Leu	0.0	0.41	0.17	γ	β-strand	56.5	4.10E−06
p.Met290Val	**2.2**	0.71	**0.74**	γ	β-strand	56.5	1.23E−05
p.Val293Met	0.6	**−5.35**	**1.33**		β-strand	0.6	1.80E−05
p.Gly294Ala^▲^	−0.2	**−1.01**	0.23	γ	coil	54.5	2.15E−05
p.Gly294Arg^▲^	**−1.0**	**−1.01**	−0.02	γ	coil	54.5	1.21E−05
p.Lys299Asn	**2.5**	**−1.31**	−0.26		turn	36.5	5.69E−05
p.Arg301His^■^	**1.3**	−0.79	**0.84**	γ, knob	turn	35.0	8.00E−06
p.Ala305Gly^▲^	0.6	−0.93	**0.71**	γ	β-strand	41.5	3.99E−06
p.Tyr306Cys^■^	**1.2**	**−6.61**	**0.81**	γ	β-strand	61.0	3.19E−05
p.Tyr306His^▲^	**1.3**	**2.54**	0.30	γ	β-strand	61.0	3.99E−06
p.Ala308Val	0.9	−0.98	0.05		β-strand	16.4	3.99E−06
p.Ala308Thr	**1.7**	**2.44**	0.17		β-strand	16.4	5.67E−05
p.Gly309Asp	**2.3**	−0.64	**1.42**		β-strand	71.2	3.98E−06
p.Asp314Asn	−0.2	−0.51	0.26		coil	27.1	7.97E−06
p.Gly322Ser	**3.7**	−0.03	**0.56**		turn	79.6	7.96E−06
p.Asp323Asn	−0.8	**1.89**	0.13	Knob	turn	65.5	3.98E−06
p.Asp324Glu	0.0	0.26	**0.82**	γ, knob	turn	67.9	1.19E−05
p.Phe330Leu	0.4	−0.92	**0.90**	γ, knob	α-helix	30.7	3.98E−06
p.Asn334Ile^■^	0.5	−0.18	−0.04	γ	turn	46.6	1.59E−05
p.Met336Leu^▲^	−0.6	−0.84	**0.98**	γ	bridge	13.0	3.98E−06
p.Asn343Asp	0.6	−0.03	**0.83**	proximal to Ca^2+^	bridge	32.2	3.98E−06
p.Asp344Glu^▲^	0.4	−0.48	**1.01**	Ca^2+^	turn	37.3	3.98E−06
p.Lys347Thr	**2.2**	0.89	−0.18	γ, proximal to Ca^2+^	coil	69.5	3.98E−06
p.Ala367Thr^■^	**3.2**	0.39	**1.25**		turn	0.0	4.24E−05
p.Gly377Val^▲^	**4.5**	**−1.64**	**2.14**		turn	92.1	3.89E−05
p.Gly377Cys^▲^	**3.1**	0.57	**1.28**		turn	92.1	3.98E−06
p.Gly377Ser^■^	**3.1**	−0.55	**1.49**		turn	92.1	1.42E−05
p.Pro386Ser	**1.2**	**−1.97**	0.39		turn	93.4	1.99E−05
p.Asn387Lys^■^	−0.3	**2.27**	0.59	proximal to knob	turn	83.8	2.39E−05
p.Asn387Ser^▲^	**1.0**	0.24	−0.10	proximal to knob	turn	83.8	3.98E−06
p.Ile393Met	0.2	−0.23	**1.41**		coil	0.9	3.98E−06
p.Met405Val	**3.9**	**−4.26**	**0.95**		coil	0.8	3.18E−05
p.Met410Ile	**2.4**	**5.62**	0.06		β-strand	0.8	1.41E−05
p.Met410Val	**3.4**	**5.50**	**0.59**		β-strand	0.8	1.41E−05
p.Ile412Thr	**2.2**	**−10.75**	**2.93**		β-strand	3.5	3.98E−06
p.Ile413Val	0.7	**−1.83**	**1.39**		β-strand	0.0	3.98E−06
p.Pro414Thr	**3.1**	**−3.98**	**1.45**		β-strand	11.3	1.19E−05
p.Asn416Asp	−0.3	**1.33**	−0.18		turn	95.9	3.19E−05

gnomAD amino acid variants, ∆∆Gs (calculated with FoldX, CUPSAT, and PoPMuSiC on the crystal structure of the C-terminal fragment of the fibrinogen gamma chain monomer, PDB 3FIB), molecular interactions, protein secondary structure, and solvent-accessible surface of the residues affected by mutations, and gnomAD allele frequency. ^a^ Intermolecular interactions of the wild type residues with functional ligands (Bβ, γ, knob, and Ca^2+^ ion, determined in the crystal structure of fragment double-D from human fibrin, PDB 1FZC). ^b^ Protein secondary structure of the wild type residues (from PDB 3FIB). ^c^ Percentage of the solvent-accessible surface of side chains of the wild type residues (determined on PDB 3FIB). ^d^ Structurally significant ∆∆Gs are in bold. ∆∆Gs are enclosed in dashed boxes if, for a given variant, significant structural effects are predicted by all three methods (FoldX, CUPSAT, and PoPMuSiC). For both FoldX and CUPSAT, mutations with destabilizing and stabilizing effects on protein structure are respectively associated with ∆∆G > 0 and ∆∆G < 0, while for PoPMuSiC is the inverse, i.e., ∆∆G > 0 for stabilizing mutations and ∆∆G < 0 for destabilizing ones. The ∆∆G thresholds above which mutations are assumed to produce significant structure alteration are as follows: for both FoldX and CUPSAT, |∆∆G| > 1.0 Kcal/mol, while for PoPMuSiC, |∆∆G| > 0.5 Kcal/mol (see Materials and Methods for references on these thresholds and the predictive accuracies of the individual methods on experimentally determined mutations). ^e^ Mean and standard deviation of all ∆∆Gs calculated by the individual methods. ^■^ Same variant is also reported in the pathogenic HGMD database. ^▲^ A variant hitting the same residue but with different substituting amino acids is reported in HGMD.
